# 

**Published:** 1906-06

**Authors:** 


// THE AMERICAN
### Journal of Dental Science

**Founded, Owned, Edited and Published by the Dental Profession of America.**

**THIRD SERIES.**

**VOLUME XXXVII. JUNE, 1906. NO. 6.**

EDITORS:

WM. GIRD BEECROFT, D. D. S., Madison, Wis. B. H. TEAGUE, D. D. S., Aiken, S. C

Published on the 10th day of each month
Subscription Price, $1 00 per year. Foreign countries, $1.50 per year.

*Entered as Second-Class Matter, Madison, Wis.*

Address all communications, both business and editorial, to
**The Dental Publishing Company, Madison, Wisconsin.**

### SOCIETY ANNOUNCEMENTS, ETC.

South Carolina State Dental Association will meet in annual session at Charleston on the Isle of Palms, June 26th, 27th, 28th and 29th. A cordial invitation is extended to all ethical dentists.

Maine Dental Society holds its forty-first annual meeting at Kineo House, Moosehead Lake, Maine, July 17th, 18th and 19th.

Kentucky State Dental Association. The time and place for the annual meeting of this association has been changed to Louisville, during home coming week, June 12th, 13th and 14th. Special railroad rates of one fare for the round trip can be obtained. All former residents of Kentucky in the profession are especially invited.

Kentucky State Board of Dental Examiners meets at Louisville, June 5th. All applicants will be examined in regular college branches. Practical work, gold and amalgam. One piece of metal work, materials, small instruments and engine must be furnished by the candidate. The fee is $20.00.

J. Richard Walton,

*President.*

The Masonic, Louisville, Ky.

Georgia State Dental Society meets in Savannah, June 19th, 20th, and 21st.

North Carolina Board of Dental Examiners. Meeting for the examination of applicants will be held at High Point, June 18th, 19th and 20th. Written examination in all regular college branches together with practical work in both operative and mechanical dentistry. Only graduates will be admitted to the examination and each applicant must bring his diploma.

R. H. Jones,
*Secretary.*

Winstom, Salem, N. C.

The Semi-Centennial Meeting of the Michigan State Dental Association will be held in Detroit, July 9, 10, 11, 1906. A most cordial invitation is extended to all reputable practitioners to attend this Fiftieth Anniversary of the Association, and a most interesting program is in preparation to celebrate this important occasion.

---

### SCRAP PILE WANTED.

Under the heading of the "Scrap Pile" I desire to make a suggestion regarding raising a fund for San Francisco sufferers. From my experience during the past few weeks, the task of acquiring a sum sufficiently large to materially assist those in need, is a difficult one, and the "Scrap pile" idea evolved from the ease with which a goodly sum was collected in Kansas City a year ago for an event of minor importance, by simply requesting all asked to donate a portion of their gold scraps, several hundred pennyweight soon accumulated.

Now, let every dentist regardless of the fact whether or no, he has paid into other funds, box up a portion of his gold scraps, and send them to Dr. J. D. Patterson of Kansas City, Mo., Keith & Perry Bldg., Treasurer of National Relief Committee, and further assist those in need.

Yours,

J. P. Root.

---

The enterprise and courage of the members of the San Francisco drug trade were clearly exemplified during the recent disaster. Before the fire was extinguished they placed large orders with the manufacturing chemists. One house ordered

30,000 pounds of antiphlogistine, and altogether over 100,000 pounds were shipped to the coast upon order within a week.

On a steamer from New York, running up the California coast at the time of the earthquake, were 35,000 pounds of antiphlogistine, and upon orders from the home office, the emergency hospitals were liberally supplied free of charge.

### "To Check Hemorrhage at Once in Cases of Pulp Removal"—Use Dr. Veo's Remedy.

Take a fine, smooth broach, wind a little cotton on the end, moisten with REMEDY. When pulp is removed, carry the REMEDY into the root canal and leave it for a few minutes,—THERE WILL BE NO HEMORRHAGE. Often it is best to leave REMEDY on small piece of cotton in root canal at least a week if tooth is sore or sensitive. Before filling the root canal, wash it out with absolute alcohol.

### To Our Subscribers.

The wind bloweth, the water floweth, the farmer soweth, and the subscriber oweth, and the good Lord knoweth that we are in need of our dues. So come a runnin' ere we go a gunnin'; this thing of dunnin' gives us the blues.

*Exchange.*

